# Actuation delay compensation of robots in semi-physical test

**DOI:** 10.3389/fnbot.2022.1099656

**Published:** 2023-01-09

**Authors:** Xiao Zhang, Yun He, Zhigang Xu, Zainan Jiang, Yong Liu, Wenbo Feng, Junwu Wu

**Affiliations:** ^1^Shenyang Institute of Automation, Chinese Academy of Sciences, Shenyang, China; ^2^Institutes for Robotics and Intelligent Manufacturing, Chinese Academy of Sciences, Shenyang, China; ^3^State Key Laboratory of Robotics and Systems, Harbin Institute of Technology, Harbin, China; ^4^University of Chinese Academy of Sciences, Beijing, China; ^5^Institute of Aerospace System Engineering Shanghai, Shanghai, China

**Keywords:** semi-physical test, actuation delay compensation, docking test platform, space docking dynamics modeling, robotic systems

## Abstract

In general, the traditional spacecraft semi-physical docking tests include the evaluation of docking and separation performance. However, these tests often rely on “specific” equipment, such as specially designed actuators and fast-response hydraulic systems, to meet the stringent dynamic response requirements of semi-physical testing. In this paper, a novel docking test platform is designed based on a general-purpose industrial manipulator using 3-D force and 3-D torque sensors. Different from the traditional solution, this novel platform is well-assembled and cost-effective. Furthermore, an actuation delay compensation method is introduced to improve the performance. Finally, the proposed method is evaluated using simulations. The results show that the novel method is with promising performance in terms of actuation delay compensation.

## 1. Introduction

The space docking mechanism plays a role in realizing the docking of spacecraft in space under various forms of initial docking conditions and then fulfilling better functions. To ensure the progressive development of docking in space, many tests must be conducted on the ground. The ground test of the space docking mechanism goes beyond its mechanical characteristic test and docking performance test. The docking performance test generally needs to reproduce the working process of the docking mechanism in space on the ground. This involves the application of common spacecraft ground tests to overcome gravity (Xu et al., 1994, Carignan and Akin, 2000, How et al., 2001). For example, the space manipulator can accomplish many dexterous tasks in space (Jiang et al., 2019a,b), but in the ground test, the test equipment plays a major role in balancing the weight of the space manipulator itself. In the process of testing the docking mechanism on the ground, the spacecraft matters in simulating the load of the docking mechanism. One commonly used method is to simulate the mass and inertia of the spacecraft in an entirely physical way and then compensate for the gravity of the simulated spacecraft through air flotation. This method features high test accuracy, simpleness, and reliability in theory. Generally, the application of air flotation is achieved in a two-dimensional plane. Three-dimensional rotating air flotation can be realized by using an air flotation ball, and vertical air flotation depends on an air flotation guide column combined with an air flotation pulley. However, the design of a full air flotation mechanism cannot be achieved with six degrees of freedom. Moreover, changing the mass and inertia characteristics of the simulated spacecraft takes a lot for the full physical air flotation equipment, and so does the setting of the first-order initial conditions of docking. In this way, the beauty of semi-physical testing is reflected (Doyon et al., 2003, Zebenay et al., 2015). The semi-physical test employs the combination between a mechanical motion system and mathematical algorithm to simulate the real docking movement, which harbors the flexibility of digital simulation and the authenticity of full physical simulation. In the mathematical model, the modification of the mass and inertia characteristics of the spacecraft can be easily achieved by the operator; the implementation of the initial docking conditions is effortless for the actuator, and in addition to the mass and inertia characteristics of the spacecraft, other dynamic factors can also be added in the process of designing the mathematical model.

## 2. Our choice

To reproduce the collision process in space and accurately simulate the dynamic characteristics of objects with the tested device, the motion simulator should be equipped with a series of factors such as a high degree of freedom, high load bearing, high precision, and high dynamic response. The above characteristics are not born for serial mechanisms, which are the merits of parallel mechanisms. Therefore, at present, the motion simulators with high demand in these aspects all use parallel mechanisms, most of which are Stewart parallel mechanisms. For example, the collision ground simulation experiment system built by Boeing Company of the United States (Motaghedi and Stamm, 2005), and the collision ground simulation experiment system of McDonnell Douglas Aerospace Department of the United States (Ananthakrishnan et al., 1996). Realizing high performance of ordinary robots is one of the core problems in robotic research (Chen and Qiao, 2020a,Qiao et al., 2022b). With the development of robot application and the combination of deep learning technology and robots (Qi and Su, 2022), robots are increasingly intelligent (Chen and Qiao, 2020b,Wang et al., 2022) and behaving more and more like human beings (Su et al., 2022b). Now robots can be driven by humans to display natural and appropriate behaviors in social scenes (Liu et al., 2022a). Cylinder-aperture ESM measurement system sees the successful application of a 6-DOF manipulator (Liu et al., 2022b). Such as compliant and precise manipulation, fast and flexible response, and deep collaboration between humans and robots become possible (Qiao et al., 2022a). Even in unknown physical interaction between the patients’ body and surgical tool in laparoscopic surgery, a task-space control approach based on fuzzy approximation is proposed, so a serial redundant robot manipulator (7 degrees of freedom) could accomplish the task (Su et al., 2022a). It seems feasible for semi-physical test of space docking mechanisms to use serial robots. This paper, a set of the semi-physical docking test platforms is built through a KUKA manipulator, a three-dimension-force and three-dimension-torque sensor (F/T sensor), a PXI computer, a PC and other accessories. The existence of actuation delay in this test platform boosts the docking performance of the semi-physical test. It well managed the problem of semi-physical experiments with the low-cost serial robot scheme. Compared with the semi-physical test bench developed by the Institute of Aerospace System Engineering Shanghai and Shanghai Jiao Tong University, the cost of this test bench is only about two hundred thousand dollars, while theirs is about 2 million dollars. The bearing capacity of the test bench in this paper is on the order magnitude of 5000 N and 700 Nm, which is the same as that of their test bench. Our control cycle is 4 ms, and theirs 1 ms. When the control cycle falls behind, the algorithm designed by us fills the hardware performance gap and conducts semi-physical testing. The main contributions of our work are twofold. First, the dynamic model with the equivalent resistance of the space manipulator in parking docking of the semi-physical test is established, and the implementation method on the manipulator is found. Second, the actuation delay compensation method is available for manipulator’s lagging phenomenon in the semi-physical docking test.

The following parts are arranged as follows: Section “Semi-physical docking performance test” is the semi-physical test with modeling of space docking dynamics on the general test platform. Section “Actuation delay compensation” discusses how we find the delay effect in the test system and how to compensate for it. Additionally, the simulation studies the lagging compensation method. Section “Conclusion” is the summarization of the paper.

## 3. Semi-physical docking performance test

In this chapter, we will introduce how to implement traditional semi-physical tests with the equipment in this paper. The two key actuators of this test platform are a common industrial manipulator and an F/T sensor. The realization of all functions on the test platform is based on the flexible mechanical motion control of the manipulator and the accurate F/T sensor measurement data.

### 3.1. Semi-physical test

**FIGURE 1:**
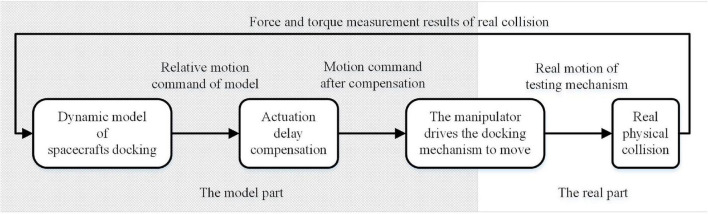
Semi-physical docking performance test structure.

The structural block diagram of the semi-physical test is shown in Figure 1.

The principle of the semi-physical test is to calculate the motion of the spacecraft under the action of an external force according to the spacecraft dynamic model and then execute the motion through the real mechanism. When the docking mechanism moves according to the results given by the dynamic model, an actual collision appears, and the sensor detects the real collision force and torque. The force and torque data are sent to the dynamic model, and the model calculates the motion of the spacecraft in the next cycle. The manipulator continues to drive the docking mechanism according to the model’s results, and the docking mechanism processes a real collision again. In this way, the iteration constitutes a semi-physical test. Theoretically, we need the manipulator to act corresponding movement as soon as the impact force and torque are generated. However, in fact, the measurement of the impact force and torque, the calculation of the mathematical model, and the execution of the manipulator’s movement all take time, which leads to the lagging effect and significantly affects the semi-physical test. The traditional method alleviates the lagging effect by improving the response speed of the actuator. In this paper, under the condition that the existence of the lagging effect lasts, a method of actuation delay compensation is proposed, and it will be discussed in the next chapter.

### 3.2. Docking dynamics model

The semi-physical model in this paper is as follows. The model describes the relationship between the force and torque of the spacecraft and the acceleration and angular acceleration during parking docking with manipulator assistance. The definition of parking docking is that two spacecraft are connected by a manipulator or other components, and the initial conditions of docking are almost static.


$${\text{F}}_{K,Q}-{\text{F}}_{s-\mathrm{manipulator}}=m_{K,Q}\,{\text{a}}_{K,Q}$$

$${\text{T}}_{K,Q}-{\text{T}}_{s-\mathrm{manipulator}}={\text{I}}_{K,Q}\,\alpha_{K,Q}+\omega_{K,Q}\times {\text{I}}_{K,Q}\,\omega_{K,Q}$$

where ***F***_K,Q_ and ***T***_K,Q_ are the measurement results of the docking force and torque, ***F***_s−*manipulator*_ and ***T***_s−*manipulator*_ are the equivalent resistances of the space manipulator in parking docking, and the mass *m*_K,Q_ and inertia tensor ***I***_K,Q_ of the spacecraft are the parameters set by the operator. Angular velocity ω_K,Q_ is a variable involved in iterative calculation. Acceleration ***a***_K,Q_ and angular acceleration α_K,Q_ are variables to be solved.

**FIGURE 2:**
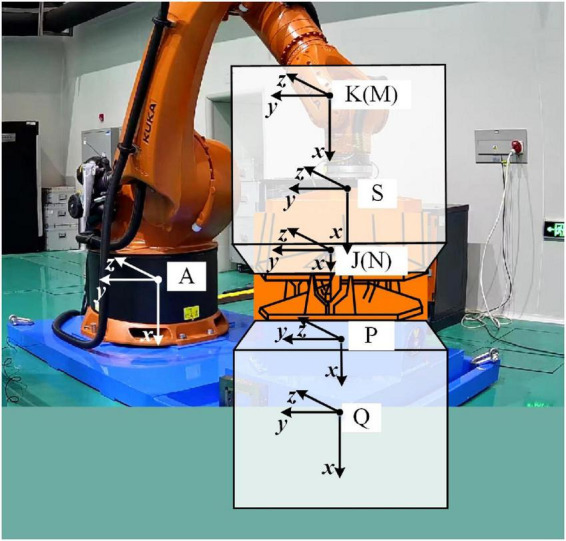
Schematic diagram of the semi-physical test with the coordinate system.


As shown in Figure 2, we imagine that there is spacecraft behind the docking mechanisms. In fact, they do not exist, and only the docking mechanisms are real. The coordinate systems of spacecraft centroids and the coordinate systems of docking mechanisms are also drawn in the figure, where A is the ground coordinate; S the F/T sensor coordinate system; K the passive spacecraft centroid, which is moveable during the test and is also named M, meaning that all the moving commands describe the movement between M and A; J the passive docking mechanism docking surface, whose positional relationship with K is invariant and is also named N, an additional observation coordinate system set by the operator; P the active docking mechanism docking surface; and Q the active spacecraft centroid. P and Q are static during the test. In the real docking process in the universe, K and Q are both moving. In the experiment, Q is stationary, and K’s motion reproduces K’s relative motion with respect to Q. The F/T sensor can measure the force and torque in the S coordinate system in real time. In the test, we need to conduct gravity compensation, zero compensation, and dynamic compensation for the measurement results and convert the compensated measurement results to the M coordinate system for the model. The compensation and conversion are shown in the following equations.

{F(compensation)⁢(n)S=F(n)S-R(n)GS⋅GG-F0S-m⁢aST(compensation)⁢(n)S=T(n)S-(R(n)GS⋅TGG+pSGS×GS)-T0S-(IS⁢αS+ωS×IS⁢ωS)

{F(n)M=R(n)SM⋅F(compensation)⁢(n)ST(n)M=R(n)SM⋅T(compensation)⁢(n)S+pMSM×F(n)M

where R(n)GS is the rotation matrix between the S coordinate system and the gravity direction coordinate system and R(n)SM is the rotation matrix between the M coordinate system and the S coordinate system. R(n)GS⋅GG and R(n)GS⋅TGG+pSGS×GS are the gravity compensation items; ${\text{F}}_{0}^{S}$ and ${\text{T}}_{0}^{S}$ are the zero compensation items; m**a**^S^ and **I**^*S*^α*^S^* + ω*^S^*×**I**^*S*^ω*^S^* are the dynamic compensation items. The parameters **G**^*G*^, pSGS, ${\text{F}}_{0}^{S}$, and ${\text{T}}_{0}^{S}$ used in gravity compensation and zero compensation can be obtained by the least square method under the condition that R(n)GS, ${\text{F}}_{\left(\mathrm{de}-\mathrm{gravity}\right)\,\left(n\right)}^{S}$, and ${\text{T}}_{\left(\mathrm{de}-\mathrm{gravity}\right)\,\left(n\right)}^{S}$ are known. The parameters m and **I**^*S*^ can also be obtained by the least square method under the condition that **a**^*S*^, α*^S^*, and ω*^S^* are known. In this paper, dynamic compensation is ignored; according to the large mass ratio between the docking mechanism and the spacecraft, only gravity compensation and zero compensation are programmed.

The F/T in the K coordinate system is directly measured, and the force in the Q coordinate system needs to be calculated according to the following equation. C is the reference coordinate system set by the operator.


$${\text{F}}_{\text{Q}}^{C}=-{\text{F}}_{\text{K}}^{C}$$

TQC=-TKC+pCQK×FKC

The acceleration, angular velocity and angular acceleration can be solved according to the method given in the following equation.


a=F/m

$$\alpha_{1}=I\backslash \left(\text{T}-\omega \times I\,\omega \right)$$

$$\alpha_{2}=I\backslash \left(\text{T}-\left(\omega +0.5\,\alpha_{1}\cdot T\,\_\,s\right)\times I\,\left(\omega +0.5\,\alpha_{1}\cdot T\,\_\,s\right)\right)$$

$$\alpha_{3}=I\backslash \left(\text{T}-\left(\omega +0.5\,\alpha_{2}\cdot T\,\_\,s\right)\times I\,\left(\omega +0.5\,\alpha_{2}\cdot T\,\_\,s\right)\right)$$

$$\alpha_{4}=I\backslash \left(\text{T}-\left(\omega +\alpha_{3}\cdot T\,\_\,s\right)\times I\,\left(\omega +\alpha_{3}\cdot T\,\_\,s\right)\right)$$

$$\alpha =\left(\alpha_{1}+2\,\alpha_{2}+2\,\alpha_{3}+\alpha_{4}\right)/6$$

In this way, we can calculate the angular velocity and angular acceleration of the active spacecraft and passive spacecraft in the model according to the measured force and torque in each motion period. The initial position, initial velocity and initial angle in the dynamic model are set by the operator. In each motion period, we calculate the attitude, velocity and angular velocity at the end of this period according to the following formula, which is used as the initial condition for the motion calculation of the next cycle.


$${\text{v}}_{K,Q\,\left(n\right)}={\text{v}}_{K,Q\,\left(n-1\right)}+\left({\text{a}}_{K,Q\,\left(n\right)}+{\text{a}}_{\mathrm{ARM}\,\left(n\right)}\right)\cdot T\,\_\,s$$

$$\omega_{K,Q\,\left(n\right)}=\omega_{K,Q\,\left(n-1\right)}+\left(\alpha_{K,Q\,\left(n\right)}+\alpha_{\mathrm{ARM}\,\left(n\right)}\right)\cdot T\,\_\,s$$

T(n)K,QC=K,QCT(n-1)TK′,Q′K(0.5(vK,Q⁢(n-1)+vK,Q⁢(n))⋅

$$T\_s,0.5\left(\omega_{K,Q\,\left(n-1\right)}+\omega_{K,Q\,\left(n\right)}\right)\cdot T\_s)$$

The acceleration ***a***_*ARM*(n)_ and angular acceleration α_*ARM*(n)_ generated by the force of the space manipulator on the spacecraft are involved in the model calculation here. They are two accelerations set by the operator. Thus far, we have completed the dynamic model of the docking of two spacecraft, both of which are moving. Provided that the test platform in this paper only allows the passive part of the docking mechanism to move and the active part of the docking mechanism to be fixed on the base, we also need to calculate the relative motion between the two docking mechanisms. Then, the manipulator performs the relative motion to complete the equivalent test. The motion of the K coordinate system relative to the Q coordinate system is calculated according to the following equation.


v(n)KQ=vK⁢(n)-vQ⁢(n)-ωQ⁢(n)×pKQ

ω(n)KQ=ωK⁢(n)-ωQ⁢(n)

Finally, we obtain the movement command of the manipulator in the following equation:


T(n)MC=T(n-1)MCTM′M(0.5(v(n-1)KQ+v(n)KQ)⋅

T_s,0.5(ω(n-1)KQ+ω(n)KQ)⋅T_s)

The initial position and velocity of the K coordinate system relative to the Q coordinate system are calculated according to the initial position and velocity of the docking dynamic model, and the manipulator is driven by the operator to reach that position. When the manipulator reaches the initial conditions, we can start the semi-physical test by switching the control mode of the manipulator to the semi-physical simulation mode.

Thus far, we have completed the dynamic model used in the semi-physical test. The model can output the motion velocity command for coordinate M.

## 4. Actuation delay compensation

### 4.1. Existence of actuation delay

**FIGURE 3:**
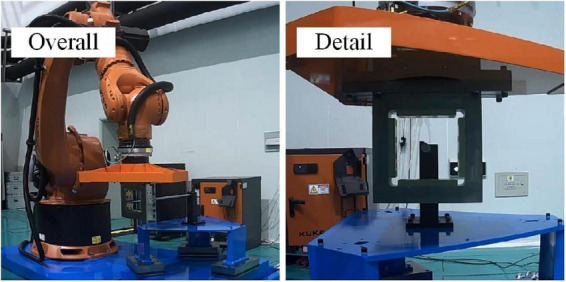
Spring rod test.

After obtaining the completed semi-physical test model, we designed a small experiment on the test equipment to verify the implementation of the semi-physical test. The designed spring rod test is shown in Figure 3.

**FIGURE 4:**
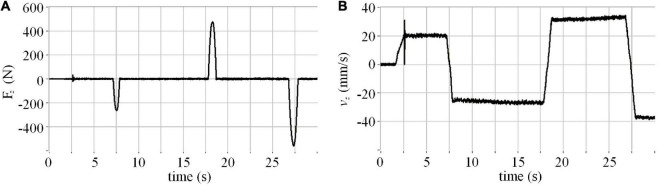
Rod test results: **(A)** Force; **(B)** velocity.

In the spring rod test, the two spacecraft theoretically did a pure elastic collision, which means the impact force of each adjacent two times should be equal in size and opposite in direction. In fact, however, we obtain the results in Figure 4.

The results demonstrate that the impact force increases with impact times. It can be inferred that in the process of impact, the movement of the manipulator, F/T measurement, computer simulation calculation, communication, and other factors lead to the lagging effect in the semi-physical test system, resulting in a longer contact time of the elastic rod and a larger rebound velocity.

### 4.2. Compensation

After many attempts, we have found a way to reduce the actuation delay of this system. We add two items to the output velocity of the model and then drive the manipulator with the final velocity as the command. To describe the way we do, we define four velocities: manipulator velocity command ***v***_*cmd*(n)_ and ω_*cmd*(n)_; dynamic model output velocity ***v***_*mod*el(n)_ and ω_*model*(n)_; compensation velocity ***v***_*comp*(n)_ and ω_*comp*(n)_; back velocity ***v***_*back*(n)_ and ω_*back*(n)_.

The relationship among these four velocities is:


$${\text{v}}_{\mathrm{cmd}\,\left(n\right)}={\text{v}}_{\mathrm{mod}\text{el}\,\left(n\right)}+{\text{v}}_{\mathrm{comp}\,\left(n\right)}+{\text{v}}_{\mathrm{back}\,\left(n\right)}$$

$$\omega_{\mathrm{cmd}\,\left(n\right)}=\omega_{\mathrm{model}\,\left(n\right)}+\omega_{\mathrm{comp}\,\left(n\right)}+\omega_{\mathrm{back}\,\left(n\right)}$$

This means that without compensation, we should use the model output velocity as the command velocity; however, in fact, we added something else to the model output velocity as the command velocity.

The definition of the dynamic model output velocity is:


vmodel⁢(n)=0.5⁢(v(n-1)KQ+v(n)KQ)

ωmodel⁢(n)=0.5⁢(ω(n-1)KQ+ω(n)KQ)

To describe the compensation velocity, the following variables are defined. The velocity difference dv_(n)_, dω_(n)_ lies in the difference between the current cycle model velocity and the previous cycle model velocity.


$${\text{dv}}_{\left(n\right)}={\text{v}}_{\mathrm{model}\,\left(n\right)}-{\text{v}}_{\mathrm{model}\,\left(n-1\right)}$$

$$\text{d}\,\omega_{\left(n\right)}=\omega_{\mathrm{model}\,\left(n\right)}-\omega_{\mathrm{model}\,\left(n-1\right)}$$

Remark: It should be noted that signals ***v***_*model*(n)_ and ω_*model*(n)_ have gone through another data preprocessing, which is a combination of a low-pass filter and saturation.

The definitions of velocity difference symbols ***s***_*dv*(n)_ and ***s***_dω(n)_ are judging each component of the velocity vector and angular velocity vector. When the absolute value of the component is less than the set threshold ***v***_*min*⁡dv_, ω_*min*⁡dω**v**_, the component becomes 0. When the absolute value of the component is greater than the set threshold, the symbol of the component is defined as the component. That is, if the component is positive, the value is 1, and if the component is negative, or the value is −1.


$${\text{s}}_{\text{dv}\,\left(n\right)}\,\left(m\right)=\left\{ \begin{array}{c} 0,\mathrm{when}\,\left|{\text{dv}}_{\left(n\right)}\,\left(m\right)\right|<v_{\min\text{dv}}\\ \mathrm{sign}\,{\text{dv}}_{\left(n\right)}\,\left(m\right),\mathrm{when}\,\left|{\text{dv}}_{\left(n\right)}\,\left(m\right)\right|\geq v_{\min\text{dv}} \end{array}\right.$$

$$s_{\text{dω}\,\left(n\right)}\,\left(m\right)=\left\{ \begin{array}{c} 0,\mathrm{when}\,\left|{\text{dω}}_{\left(n\right)}\,\left(m\right)\right|<\omega_{\min\text{dω}\,v}\\ \mathrm{sign}\,{\text{dω}}_{\left(n\right)}\,\left(m\right),\mathrm{when}\,\left|{\text{dω}}_{\left(n\right)}\,\left(m\right)\right|\geq \omega_{\min\text{dω}\,v} \end{array}\right.$$

The velocity symbols ***s***_v(n)_ and ***s***_ω(n)_ are:


$${\text{s}}_{v\,\left(n\right)}=-\mathrm{sign}\,{\text{v}}_{\mathrm{model}\,\left(n\right)}$$

$${\text{s}}_{\omega \,\left(n\right)}=-\mathrm{sign}\,\omega_{\mathrm{model}\,\left(n\right)}$$

The compensation of velocities is defined as the following equations:


$$v_{\mathrm{comp}\,\left(n\right)}=b_{0}\,s_{\text{dv}}+b_{1}\,s_{v\,\left(n\right)}\,\left|{\text{dv}}_{\left(n\right)}\right|+$$

$$\sum_{1}^{m}\left(c_{\text{m}}\cdot \sum_{0}^{n}\left(s_{v\,\left(n\right)}\,{\left|{\text{dv}}_{\left(n\right)}\right|}^{m}\right)\right)$$

$$\omega_{\mathrm{comp}\,\left(n\right)}=h_{0}\,s_{\text{dω}}+h_{1}\,s_{\omega \,\left(n\right)}\,\left|{\text{dω}}_{\left(n\right)}\right|+$$

$$\sum_{1}^{m}\left(k_{\text{m}}\cdot \sum_{0}^{n}\left(s_{\omega \,\left(n\right)}\,{\left|{\text{dω}}_{\left(n\right)}\right|}^{m}\right)\right)$$

This is a scalar equation, which should be calculated separately for each component of all vectors. Where ***b***_0_ is the coefficient of velocity difference symbol, ***b***_1_ the proportionality factor of velocity difference and velocity symbol, ***c***_m_ the integral coefficient of velocity difference polynomial, and the right of the equation are polynomial with degree m up to four. Likewise, ***h***_0_ the coefficient of the rotating velocity difference symbol, ***h***_1_ the proportionality factor of the rotating velocity difference and rotating velocity symbol, and ***k***_m_ the integral coefficient of the rotating velocity difference polynomial. The composition of the compensation function is similar to the addition of the velocity difference polynomial and velocity difference polynomial integral. This compensation reduces the collision duration.

Then, the regression function is defined as the following equations:


$$v_{\mathrm{back}\,\left(n\right)}=k_{\text{pv}}\left(v_{\mathrm{model}\,\left(n\right)}-v_{\mathrm{cmd}\,\left(n-1\right)}\right)+k_{\text{iv}}\cdot$$

$$\sum_{0}^{n}\left(v_{\mathrm{model}\,\left(n\right)}-v_{\mathrm{cmd}\,\left(n-1\right)}\right)$$

$$\omega_{\mathrm{back}\,\left(n\right)}=k_{\text{pω}}\left(\omega_{\mathrm{model}\,\left(n\right)}-\omega_{\mathrm{cmd}\,\left(n-1\right)}\right)+k_{\text{iω}}\cdot$$

$$\sum_{0}^{n}\left(\omega_{\mathrm{model}\,\left(n\right)}-\omega_{\mathrm{cmd}\,\left(n-1\right)}\right)$$

This equation is also a scalar equation, which should be calculated separately for each component of all vectors. *k*_pv_, *k*_iv_, *k*_pω_, and *k*_iω_ are the proportionality factor and integral coefficients. The equations are PI-like controllers. With a proper combination of compensation and regression, one can allow the command velocity to overcome the actuation delay in the semi-physical test in this paper.

After compensation, the semi-physical simulation motion mode expression of the manipulator is:


T(n)MC=MCT(n-1)⁢TM′M⁢(vcmd⁢(n)⋅T⁢_⁢s,ωcmd⁢(n)⋅T⁢_⁢s)

**FIGURE 5:**
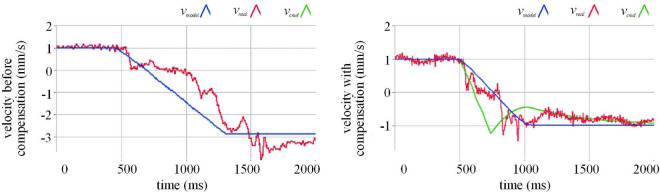
The effect of lagging compensation.

We use a single-degree-of-freedom collision experiment to observe the effect of actuation compensation. The comparison between the before and after compensation effects can be seen in Figure 5. The blue curve in the figure represents the motion speed curve calculated by the model according to the actual measured force. As a motion command, this speed can be directly output to the robot for execution, or output to the robot for execution after the compensation algorithm. We can see that before compensation, the output speed of the model changes from 1 mm/s before collision to nearly −3 mm/s after the collision, and the absolute value of the speed increases. This phenomenon does not conform to the theoretical elastic collision. In order to observe the cause of this phenomenon, we employ the red curve to draw the measured execution speed of the robot after receiving the command. It can be seen from the figure before compensation that the actual execution speed of the robot lags behind the blue movement speed command curve, and there is a short pause when the speed reaches 0. So we designed a compensation algorithm. The speed command output from the model is delayed by the compensation algorithm, and becomes a green speed command curve and output to the robot. The fact is that the compensated green speed command curve and the model output speed curve have obvious deviations at the time of the collision. After the collision, the compensated speed curve and the model output speed curve gradually coincide. The actual motion curve of the robot represented by the red curve still lags behind the speed command after compensation, which is determined by the hardware characteristics of the system. However, the red robot execution speed curve is closer to the blue model output speed curve than before compensation, indicating that the compensation algorithm makes up for the implementation lag caused by the hardware performance. At the same time, the blue model output speed curve becomes–1 mm/s after the collision, which is basically consistent with the absolute value of the speed before the collision. It can be concluded that our compensation algorithm corrects the motion distortion in the semi physical collision process, making the motion closer to the real physical characteristics.

This is consistent with our originally noticed designing functions.

### 4.3. Simulation of actuation delay compensation

**FIGURE 6:**
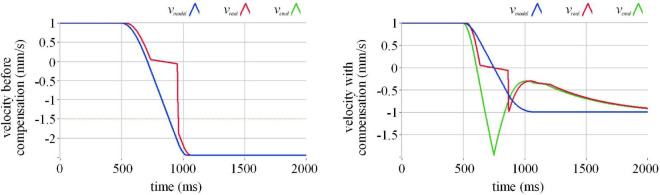
The effect of actuation compensation at velocity 1 mm/s in simulation. Where the blue line represents the command velocity, the red line the execution velocity, and the green line the command velocity after the compensation.

**FIGURE 7:**
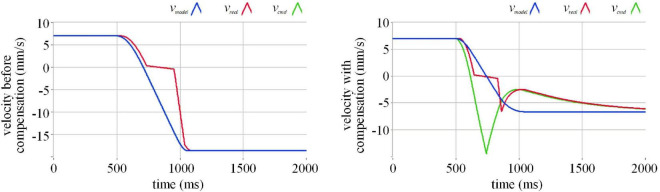
Effect of actuation compensation at a velocity of 7 mm/s in the simulation. Where the blue line represents the command velocity, the red line the execution velocity, and the green line the command velocity after the compensation.

**FIGURE 8:**
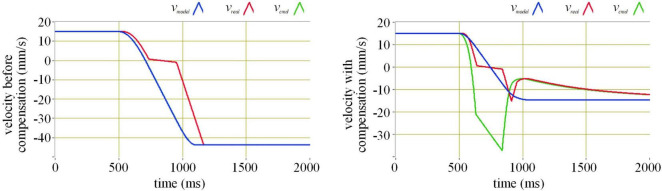
The effect of actuation compensation at velocity 15 mm/s in simulation. Where the blue line represents the command velocity, the red line the execution velocity, and the green line the command velocity after the compensation.

To study how the compensation algorithm compensates for the actuation delay of the semi-physical test, we carry out a simulation of a single-degree-of-freedom collision experiment. In the simulation, the execution velocity of the manipulator lags behind the command velocity by 32 milliseconds, and a short pause occurs when the execution velocity passes zero. The comparison of before and after compensation in the simulation of different initial velocities is demonstrated in Figures 6–8.

In the simulation, the values of the coefficients *b*_0_ = 0.1, *b*_1_ = 2, *c*_1_ = 2.57, *c*_2_ = −16.81, *c*_3_ = 146, *c*_4_ = 1337, *k*_pv_ = 0.005, and *k*_iv_ = 0.000005.

Observing the simulation results of different velocities, without compensation, the absolute value of the rebound velocity is greater than that of the incident velocity. With compensation, the absolute value of the rebound velocity is almost the same as that of the incident velocity. The actuation delay compensation performs well.

**FIGURE 9:**
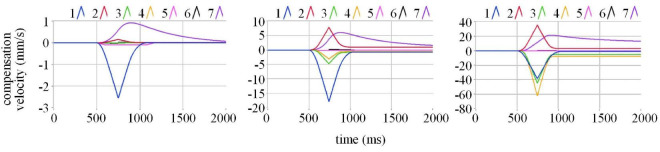
Components of the compensation velocity.

To observe the actions of each component of compensation, we draw 7 components of compensation velocity on the basis of different incident velocity conditions in Figure 9.

Where the blue line is $c_{1}\cdot \sum_{0}^{n}\left(s_{v\,\left(n\right)}\,{\left|{\text{dv}}_{\left(n\right)}\right|}^{1}\right)$, the red line is $c_{2}\cdot \sum_{0}^{n}\left(s_{v\,\left(n\right)}\,{\left|{\text{dv}}_{\left(n\right)}\right|}^{2}\right)$, the green line is $c_{3}\cdot \sum_{0}^{n}(s_{v\,\left(n\right)}$|dv_(n)_^|3^), the yellow line is $c_{4}\cdot \sum_{0}^{n}\left(s_{v\,\left(n\right)}\,{\left|{\text{dv}}_{\left(n\right)}\right|}^{4}\right)$, the pink line is *b*_0_*s*_dv_, the black line is *b*_1_*s*_v(n)_|dv_(n)_|, and the purple line is ***v***_*back*(n)_. It displays that at low velocity, the role of the blue lines $c_{1}\cdot \sum_{0}^{n}\left(s_{v\,\left(n\right)}\,{\left|{\text{dv}}_{\left(n\right)}\right|}^{1}\right)$ is obvious. With velocity increasing, the roles of the red, green and yellow lines, $c_{2}\cdot \sum_{0}^{n}\left(s_{v\,\left(n\right)}\,{\left|{\text{dv}}_{\left(n\right)}\right|}^{2}\right)$, $c_{3}\cdot \sum_{0}^{n}\left(s_{v\,\left(n\right)}\,{\left|{\text{dv}}_{\left(n\right)}\right|}^{3}\right)$, and $c_{4}\cdot \sum_{0}^{n}\left(s_{v\,\left(n\right)}\,{\left|{\text{dv}}_{\left(n\right)}\right|}^{4}\right)$ matter more. The simulation results demonstrate that within the velocity range required by the test, the actuation delay compensation method provided by us is of outstanding practice.

## 5. Conclusion

This paper represented a successful application of a set of serial robots, and realized the semi-physical test of the space docking mechanism. Compared with the semi-physical test of the space docking mechanism organized by the traditional parallel mechanism, our hardware platform does have insufficient performance, especially the implementation lag. We made up for this lag through algorithms, and achieved good results in practical applications. Based on this serial robot platform, we have finished the theoretical algorithm of space semi-physical testing, which includes the automatic calibration method of gravity compensation of sensors and the simulation model of resistance characteristics of space manipulator in docking test. The operator can set the first-order initial conditions for docking, and the application of the platform is widespread.

In the future, new semi-physical test equipment will be unfolded. This paper provides a reference solution for the problems that may be encountered during the construction of the new test bench. Our idea of compensation for actuation delay can be recognized as a reference in similar occasions where there is a demand for motion performance. The research will be ongoing and increasingly powerful and functional test equipment will be constructed in the coming days.

## Data availability statement

The original contributions presented in this study are included in the article/supplementary material, further inquiries can be directed to the corresponding authors.

## Author contributions

XZ: producing the main ideas, modeling, programming, experiments, simulation, and write the manuscript. YH, ZX, and YL: supervision, conceptualization, resources, and related ideas. ZJ: literature review and reviewing the article. WF: experiments, checking the program, and the main idea. JW: experiments, checking the program, and the modeling. All authors contributed to the article and approved the submitted version of the manuscript.

## References

[B1] Ananthakrishnan S. Teders R. Alder K. (1996). Role of estimation in real-time contact dynamics enhancement of space station engineering facility. *IEEE Robot. Autom. Mag.* 3 20–28. 10.1109/100.540146

[B2] Carignan C. R. Akin D. L. (2000). The reaction stabilization of on-orbit robots. *IEEE Control Syst. Mag.* 20 19–33. 10.1109/37.887446

[B3] Chen J. Qiao H. (2020a). Motor-cortex-like recurrent neural network and multi-tasks learning for the control of musculoskeletal systems*. *IEEE Trans. Cogn. Dev. Syst.*

[B4] Chen J. Qiao H. (2020b). Muscle-synergies-based neuromuscular control for motion learning and generalization of a musculoskeletal system. *IEEE Trans. Syst. Man Cybern. Syst.* 51 3993–4006. 10.1109/TSMC.2020.2966818

[B5] Doyon M. Piedboeuf C. Aghili F. Gonthier Y. Martin E. (2003). “The SPDM task verification facility: on the dynamic emulation in one-g environment using hardware-in-the-loop simulation,” in *Proceeding of the 7th international symposium on artificial intelligence, robotics and automation in space (i-SAIRAS 2003)*, Nara.

[B6] How J. Kitts C. Twiggs R. (2001). *A low-cost spacecraft mission for validating formation flying technologies.* Stanford, CA: Stanford University.

[B7] Jiang Z. Li Z. Li C. Yang D. Liu H. (2019a). Design and preliminary ground experiment for robotic assembly of a modular space telescope. *IEEE Access* 7 160870–160878. 10.1109/ACCESS.2019.2950666

[B8] Jiang Z. Ni F. Yang D. Li C. Yang F. Liu H. (2019b). A hybrid mapping method with position and stiffness for manipulator teleoperation. *Appl. Sci.* 9:5005. 10.3390/app9235005

[B9] Liu X. Jiang W. Su H. Qi W. Ge S. S. (2022a). “A control strategy of robot eye-head coordinated gaze behavior achieved for minimized neural transmission noise,” in *Proceedings of the IEEE/ASME transactions on mechatronics*, (Piscataway, NJ: IEEE). 10.1109/TMECH.2022.3210592

[B10] Liu X. Li X. Su H. Zhao Y. Ge S. S. (2022b). The opening workspace control strategy of a novel manipulator-driven emission source microscopy system. *ISA Trans*. Online ahead of print, 10.1016/j.isatra.2022.09.002 36163198

[B11] Motaghedi P. Stamm S. (2005). “6 dof testing of the orbital express capture system,” in *Procceding of the SPIE 5799 modeling, simulation, and verification of space-based systems II*, (Orlando, FL), 66–81. 10.1117/12.606222

[B12] Qi W. Su H. (2022). A cybertwin based multimodal network for ecg patterns monitoring using deep learning. *IEEE Trans. Indust. Inform.* 18 6663–6670. 10.1109/TII.2022.3159583

[B13] Qiao H. Chen J. Huang X. (2022a). A survey of brain-inspired intelligent robots: integration of vision, decision, motion control, and musculoskeletal systems. *IEEE Trans. Cybern*. 52 11267–11280. 10.1109/TCYB.2021.3071312 33909584

[B14] Qiao H. Zhong S. Chen Z. Wang H. (2022b). Improving performance of robots using human-inspired approaches: a survey. *Sci. China Inf. Sci.* 65 1–31.

[B15] Su H. Qi W. Schmirander Y. Ovur S. E. Cai S. Xiong X. (2022b). A human activity-aware shared control solution for medical human–robot interaction. *Assem. Autom*. 42 388–394. 10.1108/AA-12-2021-0174

[B16] Su H. Qi W. Chen J. Zhang D. (2022a). Fuzzy Approximation-based Task-Space Control of Robot Manipulators with Remote Center of Motion Constraint. *IEEE Trans. Fuzzy Syst.* 30 1564–1573. 10.1109/TFUZZ.2022.3157075

[B17] Wang D. Ma G. Liu X. (2022). An intelligent recognition framework of access control system with anti-spoofing function. *AIMS Math.* 7 10495–10512. 10.3934/math.2022585

[B18] Xu Y. Brown H. B. Friedman M. Kanade T. (1994). Control system of the self-mobile space manipulator. *IEEE Trans. Control Syst. Technol.* 2 207–219. 10.1109/87.317978

[B19] Zebenay M. Boge T. Krenn R. Choukroun D. (2015). Analytical and experimental stability investigation of a hardware-in-the-loop satellite docking simulator. *Proc. Inst. Mech. Eng. G J. Aerosp. Eng.* 229 666–681. 10.1177/0954410014539290

